# Near-gapless genome of *Sassafras tzumu* provides insights into magnoliid evolution and cellulose synthase expansion associated with wood formation

**DOI:** 10.1093/hr/uhag222

**Published:** 2026-06-03

**Authors:** Xianyin Ding, Zhuchou Lu, Yue Zhang, Tianle Xu, Yanjie Li

**Affiliations:** Research Institute of Subtropical Forestry, Chinese Academy of Forestry, Hangzhou 311400, China; Research Institute of Subtropical Forestry, Chinese Academy of Forestry, Hangzhou 311400, China; Zhejiang Yalin Biotechnology Co., Ltd, Hangzhou 311400, China; Zhejiang Forest Resource Monitoring Center, Hangzhou 310000, China; Research Institute of Subtropical Forestry, Chinese Academy of Forestry, Hangzhou 311400, China

Dear Editor,

*Sassafras tzumu* (Hemsl.) Hemsl. is an endemic tree species in East Asia with considerable ecological and economic value. It is widely used for its fine-grained and durable wood, rich aromatic oils, and ornamental value. Despite its multifaceted importance, the absence of genomic resources has hindered our understanding of its evolutionary position and the molecular basis of its wood-related traits. To construct a high-continuity reference genome, we employed a comprehensive strategy. We combined PacBio HiFi long reads, Illumina short reads for polishing, and Hi-C data for chromosome-level scaffolding. The resulting assembly spans 929.18 Mb, anchored to 12 pseudochromosomes, and achieves an impressive contig N50 of 78.46 Mb. The assembly achieves a BUSCO completeness score of 98.8%, indicating that nearly all conserved core eukaryotic genes are present and intact. To experimentally verify the genome size of *S. tzumu*, flow cytometry (FCM) was performed using a Sysmex CyFlow® Cube6 instrument. Fresh leaf tissue was processed and stained with propidium iodide following standard protocols. Phyllostachys edulis (2048 Mb) was used as the reference species. The genome size was calculated based on the ratio of the geometric mean of the fluorescence intensity between the sample and the reference. A standout feature of this genome is its exceptional continuity: 10 of the 12 chromosomes are assembled as single contigs and are entirely gapless. The remaining two chromosomes (Chr08 and Chr12) each contain only a single gap of 100 bp located in the centromeric regions. Furthermore, telomeric motifs were identified at both ends of seven chromosomes (Chr01–06 and Chr09) and at one end of the remaining five, confirming a near-complete telomere-to-telomere reconstruction. This assembly represents one of the most complete genomes within Lauraceae to date, substantially surpassing previously published genomes, such as *Cinnamomum kanehirae* [1]. It provides a robust foundation for exploring the evolution and genetic regulation of wood formation in Lauraceae and offers reliable genomic evidence to investigating the evolutionary position of magnoliids among angiosperms (Fig. 1A).

**Figure 1:**
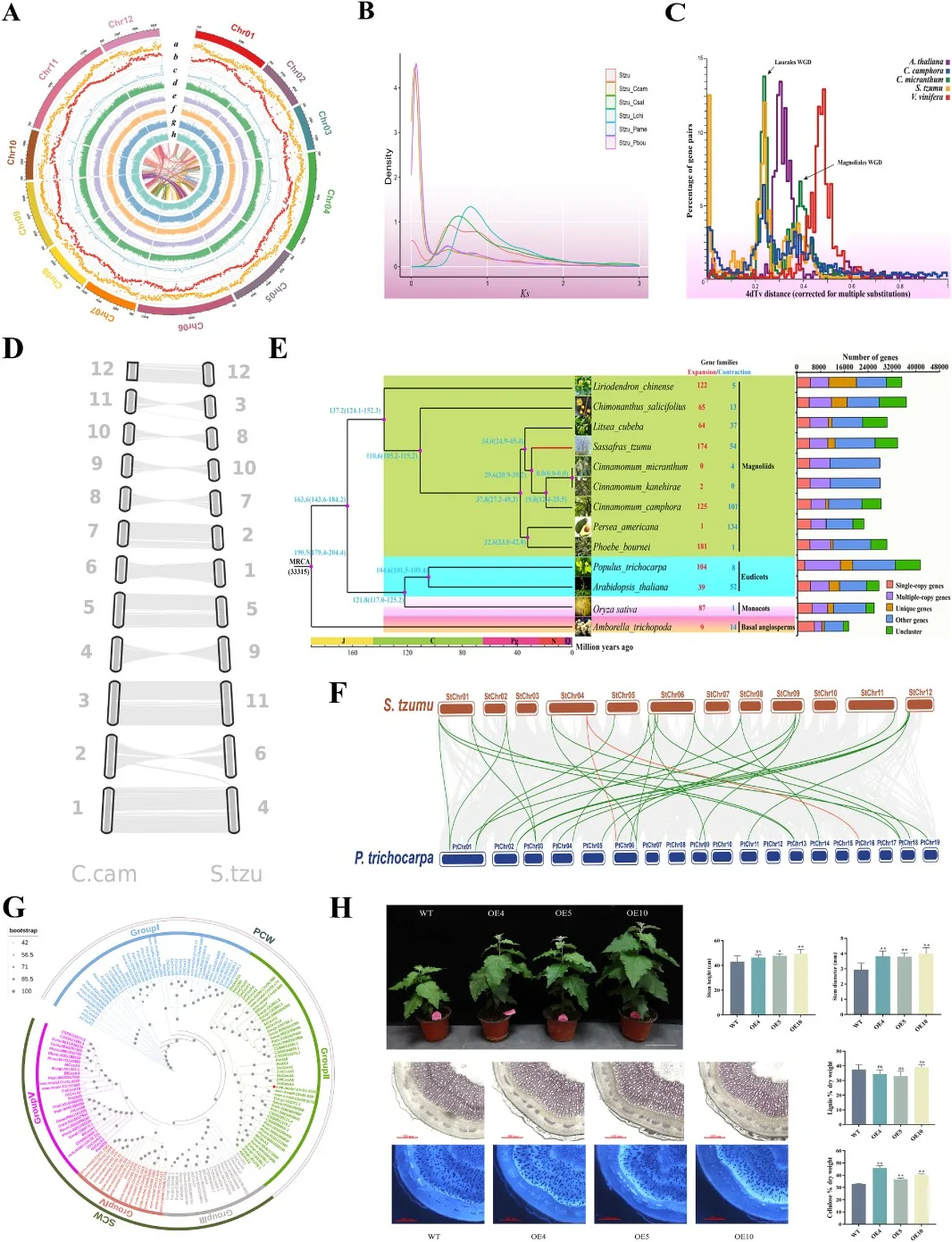
Overview of the *S. tzumu* genome, comparative genomics analysis and functional characterization of *StCesA1*. (A) Circos plot of the *S. tzumu* genome and chromosome-encoded genomic features. Each feature was calculated in 10-Mb windows across the chromosomes. Panels A–C show gene density, transposable element density, and GC content, respectively, while panels D–H depict gene expression levels in tender leaves, phloem, xylem, roots, and seeds. (B) Intergenomic *K*_s_ comparisons within Magnoliales species. The *x*-axis represents the synonymous substitution rate, and the *y*-axis is the density or frequency distribution of *K*_s_ values. (C) Comparative analysis of the 4DTv values within different species. The *x*-axis represents the 4-fold degenerate transversion rate, and the *y*-axis is percentage of 4DTv gene pairs. (D) Chromosomal synteny between *S. tzumu* and *C. camphora*. (E) Phylogenomics of magnoliids. The timeline of species divergence is represented by the Jurassic (J), Cretaceous (C), Paleogene (P), Neogene (N), and Quaternary (Q) periods. (F) Genomic collinearity analysis of *S. tzumu* and *P. trichocarpa*. The highlighted curves represent members of the CesA gene family, and the red highlighted curves represent the collinearity distribution of members of *StCesA1*. (G) Phylogenetic relationship of the CesA gene family across 10 representative species. The tree was constructed using the neighbor-joining method with 1000 bootstrap replicates. Gray circles at the nodes indicate bootstrap support values (ranging from 42 to 100), with the circle diameter proportional to the support level. The CesA members are categorized into two functional groups: those enclosed by a pink semicircle are associated with PCW biosynthesis, while those within the dark green semicircle are related to secondary cell wall development. The gene used for overexpression in this study, evm.model.Chr04.3146 (StCesA1), is highlighted with a red star. (H) Overexpression of *StCesA1* in *Populus* × *canescens*. Top: growth phenotypes (scale bar: cm). Middle: stem cross-sections stained with phloroglucinol-HCl. Bottom: cellulose fluorescence staining. Right: statistical analysis of growth and cell wall components. Error bars represent SD; asterisks indicate significant differences (^*^P < 0.05, ^**^P < 0.01). Statistical significance was assessed using a two-tailed Student’s *t*-test.

Through analyses of synonymous substitution rates (*K*_s_) and 4-fold degenerate transversion (4DTv) sites, we identified two distinct whole-genome duplication (WGD) signals: an ancient WGD commonly referred to as a magnoliid-shared WGD [2], and a more recent WGD signal observed within Lauraceae in our sampling (Fig. 1B and C). These WGD events likely fueled the diversification and environmental adaptation of the lineage. Comparative synteny analyses between *S. tzumu* and *C. camphora* revealed extensive one-to-one chromosomal collinearity (Fig. 1D), suggesting that despite the long divergence time, the overall genome structure has remained remarkably stable following these shared WGD events. Phylogenetic reconstruction based on 130 single-copy orthologs from 13 representative angiosperm species robustly supports the basal placement of magnoliids as a sister lineage to the clade comprising eudicots and monocots, consistent with the studies on *Magnolia biondii* [3]. Molecular dating analysis further estimates that *S. tzumu* diverged from other Lauraceae lineages ~29.6 million years ago (Fig. 1E). To rule out gene count inflation, we performed a comprehensive repeat annotation combining *ab initio* prediction (using LTR_FINDER, RepeatModeler, etc.) and homology-based alignment (RepeatMasker). The results reveal that LTR retrotransposons (46.31% of the genome) experienced a recent burst (~2–6 Ma), which is temporally distinct from the ancient WGD events (*K*_s_ ≈ 0.5). This confirms that the expanded gene families are not artifacts of recent transposon activity.

Beyond phylogenomics, the *S. tzumu* genome provides valuable insights into the genetic basis of wood-related traits. Of the 33 863 annotated protein-coding genes, we applied a birth–death probability model (CAFE 5, *P* < 0.05) and identified 174 significantly expanded gene families. Gene Ontology enrichment analysis revealed that these expansions are predominantly associated with biological processes related to cell wall organization, cellulose biosynthesis, and metabolic adaptation. Furthermore, genomic collinearity analysis between *S. tzumu* and *P. trichocarpa* visually illustrates the chromosomal distribution of the expanded CesA gene family, particularly highlighting the syntenic relationships of StCesA1 (Fig. 1F). Notably, the StCesA gene family exhibited the most pronounced expansion with 31 members identified in *S. tzumu*—a substantially higher count than in model woody species such as *P. trichocarpa* (17 members) and *Eucalyptus grandis* (20 members) (Fig. 1G). Detailed phylogenetic analysis classified these StCesA proteins into distinct clades, revealing that many expanded members fall into clades typically associated with primary cell wall (PCW) biosynthesis. Specifically, StCesA1 clustered closely with *Arabidopsis* AtCesA1, AtCesA3, and AtCesA10, which are essential for cellulose synthesis [4]. Transcriptomic profiling across various tissues—including tender leaves, phloem, xylem, roots, and seeds—demonstrated that *StCesA1* is highly expressed specifically in the xylem tissue.

To experimentally validate this function, we overexpressed *StCesA1* in hybrid poplar *P.* × *canescens*, a transformable woody model for secondary growth and cell wall studies. After 3 months of growth under controlled conditions, the transgenic lines exhibited marked morphological enhancements compared to wild-type plants, including an 11%–15% increase in plant height and up to a 35.8% increase in stem diameter (Fig. 1H). Biochemical analysis revealed a remarkable 39.4% increase in stem cellulose content, while lignin levels remained unchanged. Importantly, this substantial gain in cellulose did not come at the expense of lignin content, which remained statistically unchanged compared to the control. Histochemical staining of stem cross-sections further confirmed enhanced cellulose deposition without any observable abnormalities in xylem structure. These findings suggest that *StCesA1* may promote growth and cellulose accumulation in hybrid poplar under the tested conditions without significantly altering lignin content, indicating its potential value as a candidate gene for future studies of wood formation and molecular breeding.

In conclusion, our study presents a near-gapless reference genome for *S. tzumu*, providing a valuable genomic resource for comparative analyses of Lauraceae and magnoliids. The genome assembly supports phylogenomic analyses relevant to the placement of magnoliids and reveals a lineage-specific expansion of the CesA gene family that may be associated with wood formation. Functional analysis of *StCesA1* in hybrid poplar suggests a potential role in promoting growth and cellulose accumulation under the tested conditions. Together, these genomic resources and preliminary functional results provide useful candidates for future studies of wood formation and molecular breeding in woody plants [5].

## Supplementary Information

Detailed materials and methods are provided in supplementary materials. The supplementary file includes descriptions of plant materials, genome sequencing and assembly, Hi-C scaffolding, flow cytometry, genome assembly quality assessment, repeat annotation, gene prediction, phylogenetic reconstruction, divergence time estimation, WGD inference, Ks and 4DTv analyses, CAFE-based gene family expansion analysis, transcriptomic profiling, StCesA1 transformation and overexpression, cellulose and lignin measurements, biological replication, and statistical testing.

## Supplementary Material

Web_Material_uhag222

## Data Availability

All data supporting the findings of this study have been deposited in the CNGB Sequence Archive (CNSA) at https://db.cngb.org/cnsa/, under the accession number CNP0008638. Detailed materials and methods are provided in supplementary materials at https://zenodo.org/records/20438333.
