# Supplementary material for: Near-gapless genome of Sassafras tzumu provides insights into magnoliid evolution and cellulose synthase expansion associated with wood formation
Source: Hortic Res. 2026 Jun 3;13(11):uhag222. doi: 10.1093/hr/uhag222 (PMC13722869; doi:10.1093/hr/uhag222)
Supplement: Supplementary file 1 [file web_material_uhag222.zip › Supplementary Materials and Methods_Sassafras_tzumu_uhag222.docx]

**Supplementary Materials and Methods**

**Title: Near-gapless genome of Sassafras tzumu provides insights into magnoliid evolution and cellulose synthase expansion associated with wood formation**

# Materials and Methods

# 1. Plant materials and sample preparation

Samples of *Sassafras tzumu* (Hemsl.) Hemsl. were collected from mature, healthy plants maintained at the Research Institute of Subtropical Forestry, Chinese Academy of Forestry, Hangzhou, China. Fresh young leaves were collected for high-molecular-weight genomic DNA extraction using a CTAB-based protocol. For transcriptome sequencing, tissues including tender leaves, phloem, xylem, roots, and seeds were collected, immediately frozen in liquid nitrogen, and stored at -80℃ until RNA extraction. Total RNA was extracted using TRIzol reagent, followed by DNase treatment to remove genomic DNA contamination. RNA integrity and concentration were assessed before library construction.

# 2. Genome sequencing and data quality control

A combined sequencing strategy was used to generate a high-continuity reference genome. PacBio HiFi long-read sequencing was performed on the PacBio Sequel II platform in circular consensus sequencing mode, generating high-accuracy long reads with an average read length greater than 15 kb and approximately 30-fold genome coverage. Illumina short-read sequencing was performed on the NovaSeq platform using 150-bp paired-end reads, producing approximately 100-fold coverage for genome size estimation, sequence correction, and assembly quality evaluation. Hi-C sequencing libraries were generated from cross-linked leaf tissue digested with DpnII, yielding approximately 100-fold chromatin interaction data for chromosome-level scaffolding.

Raw sequencing reads were subjected to quality control using FastQC v0.12.1. Low-quality bases and adapter sequences were removed, and reads with low Phred quality scores were filtered. Only high-quality clean reads were retained for downstream analyses. The final effective data ratio exceeded 95% after filtering.

# 3. Genome size estimation by k-mer analysis and flow cytometry

Genome size was estimated using two complementary approaches. First, k-mer analysis was performed using Illumina short reads. Jellyfish was used to calculate the 17-mer frequency distribution. Genome size, heterozygosity, and repeat content were inferred from the k-mer depth distribution according to the Lander-Waterman model. The k-mer analysis estimated a genome size of approximately 988.78 Mb and a heterozygosity level of approximately 0.45%.

Second, flow cytometry was used to experimentally validate the genome size. Fresh leaf tissue of *S. tzumu* was processed and stained with propidium iodide following standard procedures. Fluorescence intensity was measured using a Sysmex CyFlow Cube6 flow cytometer. Phyllostachys edulis, with an estimated genome size of 2,048 Mb, was used as the internal reference. The genome size of S. tzumu was calculated based on the ratio of the geometric mean fluorescence intensity of the sample relative to the reference standard.

# 4. Genome assembly and Hi-C chromosome scaffolding

Initial sequence evaluation was conducted using Illumina short reads. A preliminary assembly was generated using SOAPdenovo v2.04 with K = 41 to inspect contig length distribution and GC content uniformity. The final genome assembly was generated using PacBio HiFi reads as the primary data source. Primary contigs were assembled de novo using Hifiasm v0.15. The resulting contig assembly was further scaffolded using Hi-C chromatin interaction data.

Hi-C reads were processed using the Juicer and 3D-DNA pipelines to generate interaction matrices, identify potential assembly errors, correct misjoins, and anchor contigs into chromosome-scale scaffolds. Hi-C contact maps were manually inspected to validate chromosome-level ordering and orientation. The final assembly spanned 929.18 Mb, was anchored to 12 pseudochromosomes, and had a contig/scaffold N50 of 78.46 Mb. Ten of the 12 chromosomes were assembled as single gapless contigs, whereas Chr08 and Chr12 each contained one 100-bp gap located in centromeric regions. Telomeric motifs were detected at both ends of seven chromosomes and at one end of the remaining five chromosomes, supporting near-complete chromosome reconstruction.

# 5. Genome assembly quality assessment

Genome assembly completeness and structural quality were assessed using multiple complementary metrics. BUSCO v5.2.2 was used with the embryophyta_odb10 database to evaluate gene-space completeness. The final assembly achieved a BUSCO completeness of 98.80%. CEGMA analysis showed 93.55% completeness, and the LTR Assembly Index (LAI) was 13.46, supporting reference-grade continuity. Read mapping, GC content distribution, Hi-C interaction maps, telomere detection, and chromosome anchoring statistics were also examined to evaluate assembly accuracy and chromosome-scale consistency.

# 6. Repeat annotation and LTR retrotransposon analysis

Repeat annotation combined *ab initio* prediction and homology-based searches. De novo repeat libraries were constructed using LTR_FINDER v1.07, Piler v1.0, RepeatScout v1.0.5, and RepeatModeler v2.0.1. Transposable elements, including LTR retrotransposons, LINEs, SINEs, and DNA transposons, were annotated using RepeatMasker v4.1.0. Tandem repeats were identified using TRF v4.09. Homology-based repeat annotation was performed against RepBase v26.05. The final repeat annotation showed that repetitive sequences accounted for a large proportion of the S. tzumu genome, with LTR retrotransposons representing the dominant repeat class.

To evaluate recent transposable element dynamics, LTR retrotransposon insertion times were inferred from sequence divergence between paired LTRs. The timing distribution was used to assess recent LTR activity and to distinguish repeat-driven gene count inflation from ancient WGD-related gene family expansion.

# 7. Gene prediction and functional annotation

Protein-coding gene prediction integrated homology-based, *ab initio*, and transcriptome-supported evidence. Homology-based prediction was performed using GenBLAST+A v1.0.4 and GeneWise v2.4.1 with protein sequences from closely related Lauraceae species as references. Ab initio prediction was performed using Augustus v3.4.0, GlimmerHMM v3.0.4, and SNAP v2006-07-28. RNA-seq reads from tender leaves, phloem, xylem, roots, and seeds were aligned using BLAT v36 to provide transcript evidence. Predictions from the three sources were integrated using EVidenceModeler v1.1.1, and untranslated regions and alternative splicing structures were refined using PASA v2.4.1. The final annotation identified 33,863 protein-coding genes.

Functional annotation was performed using BLASTp v2.10.1 against the Swiss-Prot, TrEMBL, and NR databases with an E-value threshold of 1e-5. KEGG v95.0 and InterProScan v5.48-83.0 were used for pathway and protein domain annotation. Non-coding RNAs were annotated separately: tRNAs were predicted using tRNAscan-SE v2.0.7, rRNAs were identified by BLAST+n searches against closely related reference sequences, and miRNAs and snRNAs were annotated using INFERNAL v1.1.3 against the Rfam v14.6 database. Non-coding RNA annotations were checked to avoid overlap with protein-coding gene models. Unless otherwise specified, software was run using default parameters.

# 8. Gene family clustering and enrichment analysis

Gene family clustering was performed using protein sequences from *S. tzumu* and 12 representative angiosperm species: *Liriodendron chinense*, *Chimonanthus salicifolius*, *Litsea cubeba*, *Cinnamomum kanehirae*, *Cinnamomum micranthum*, *Cinnamomum camphora*, *Amborella trichopoda*, *Persea americana*, *Phoebe bournei*, *Arabidopsis thaliana*, *Oryza sativa*, and *Populus trichocarpa*. OrthoFinder v2.5.5 was used to identify single-copy, multi-copy, species-specific, and shared orthologous gene families. Gene Ontology (GO) and Kyoto Encyclopedia of Genes and Genomes (KEGG) enrichment analyses were performed for S. tzumu-specific and significantly expanded gene families to infer their functional relevance.

# 9. Phylogenetic reconstruction and divergence time estimation

A total of 130 single-copy orthologous gene families were extracted from the gene family clustering results. Protein sequences were aligned using MUSCLE v5.2, and the resulting alignments were concatenated to generate a phylogenomic supermatrix. A maximum-likelihood phylogenetic tree was constructed using RaxML v. Divergence times were estimated using the mcmctree program implemented in the PAML package.

Fossil or database-derived calibration points from TimeTree were used, including *A. thaliana*-*V. vinifera* (151.6-164.6 million years ago [MYA]), *A. trichopoda*-*V. vinifera* (179.0-199.1 MYA), *A. thaliana*-*P. trichocarp*a (102.0-113.8 MYA), *C. salicifolius*-*P. bournei* (99*.*6-115.9 MYA), *P. bournei*-*P. americana* (37-70 MYA), *L. cubeba*-*S.* *tzumu* (19.7-38.5 MYA), *S. tzumu*-*C. camphora* (10.5-38.3 MYA), *P. bournei*-*L. cubeba* (37-70 MYA), and *C. salicifolius*-*L. cubeba* (99.6-115.9 MYA).

# 10. Whole-genome duplication, Ks, 4DTv, and karyotype analyses

Paralogous and orthologous gene pairs were identified using protein BLAST+ v2.16.0 searches. MCScanX was used to identify collinear blocks within the *S. tzumu* genome and between *S. tzumu* and related species. Coding sequence alignments were generated using MUSCLE with protein alignments as templates. Synonymous substitution rate (Ks) distributions and fourfold degenerate third-codon transversion (4DTv) distributions were calculated for paralogous and orthologous gene pairs to infer ancient and lineage-specific WGD signals.

Karyotype and ancestral chromosome analyses were conducted using the WGDI toolkit v0.73. Collinearity between ancestral karyotypes and the genomes of *S. tzumu* and *C. camphora* was evaluated using the -icl module with mg = 25,25. Collinear blocks were identified using the -bi module, filtered using the -c module, and visualized after ancestral karyotype assignment using the -km and -ak modules with default settings.

# 11. Gene family expansion and contraction analysis

After filtering anomalous gene families from the OrthoFinder clustering results, CAFE 5 was used to estimate ancestral gene family sizes along the phylogeny using a birth-death model. Expanded and contracted gene families were identified for each branch, and significantly expanded gene families in *S. tzumu* were defined at *P* < 0.05. Functional enrichment of expanded and contracted gene families was assessed using GO and KEGG enrichment analyses. This analysis identified 174 significantly expanded gene families in *S. tzumu*, including families enriched in cell wall organization, cellulose biosynthesis, and related metabolic processes.

# 12. Positive selection analysis

Protein sequences from the 130 single-copy orthologous gene families were aligned using MUSCLE, and corresponding codon alignments were generated from the coding sequences. Positive selection was tested using codeml in the PAML package under a branch-site model, with *S. tzumu* designated as the foreground branch and the other species used as background branches. Positively selected genes were identified using a q-value threshold of < 0.05. GO and KEGG enrichment analyses were then performed to infer the biological processes and pathways associated with positively selected genes.

# 13. Identification and phylogenetic analysis of the CesA gene family

To identify cellulose synthase (CesA) genes in the *S. tzumu* genome, the Hidden Markov Model profile of the cellulose synthase domain (PF03552) was downloaded from the Pfam database and used to search the predicted protein set with hmmsearch in HMMER v3.3.2 using an E-value threshold of 1e-5. In parallel, known CesA protein sequences from Arabidopsis thaliana were used as BLASTp queries against *S. tzumu* proteins with an E-value cutoff of 1e-5 and a maximum of five alignments per query. Candidate genes identified by both HMM and BLAST+ searches were retained, filtered to remove sequences shorter than 100 amino acids, and manually validated using the NCBI Conserved Domain Search tool to confirm the presence of the cellulose synthase domain.

For CesA phylogenetic analysis, CesA protein sequences from *A. thaliana*, *O. sativa*, *Zea mays*, *Camellia sinensis*, *P. trichocarpa*, *Eucalyptus grandis*, *Betula luminifera*, *Gossypium raimondi*i, *Phaseolus vulgaris*, and *Pinus massoniana* were included. Pairwise sequence identities were evaluated using NCBI Protein BLAST+. The CesA phylogenetic tree was constructed using IQ-TREE v2.0.3 with the neighbor-joining method and 1,000 bootstrap replicates. The resulting tree was visualized and annotated using iTOL.

# 14. Interspecific synteny analysis of CesA genes

Interspecific synteny between S. tzumu and P. trichocarpa was analyzed using MCScanX. Protein sequences from both species were reciprocally aligned using BLASTp with an E-value threshold of 1e-10 and a maximum of five alignments per query. GFF files containing chromosome identifiers, gene identifiers, start positions, and end positions were merged with BLAST+ results as MCScanX input. Collinear blocks and tandem duplications were identified, and syntenic relationships were visualized using TBtools v1.098 with chromosome length files, gene feature files, and processed collinearity outputs.

# 15. Transcriptome profiling and StCesA1 expression analysis

RNA-seq data from tender leaves, phloem, xylem, roots, and seeds were used to support gene prediction and to evaluate tissue-specific gene expression. Transcript evidence was incorporated into the structural annotation pipeline, and expression profiles were summarized across tissues. *StCesA1* (evm.model.Chr04.3146) showed high expression in xylem tissue, supporting its selection as a candidate gene for functional validation.

# 16. StCesA1 cloning, vector construction, and poplar transformation

Total RNA extraction and cDNA synthesis for *S. tzumu* were performed as described above. The amplified fragment was cloned into the PBI121 vector between the XbaI and SacI restriction sites and confirmed by full-length sequencing. The recombinant plasmid was introduced into Agrobacterium tumefaciens strain GV3101.

Because a stable genetic transformation system is not currently available for *S. tzumu*, hybrid poplar *Populus × canescens* was used as a woody model system for functional validation. *Agrobacterium*-mediated transformation of *P. × canescens* was performed using a previously reported method. Positive transgenic plants were screened and confirmed by genomic PCR and RT-qPCR. PcTUB4.1 was used as the endogenous reference gene for RT-qPCR. Three StCesA1-overexpressing lines with high expression levels (OE4, OE5, and OE10) were micropropagated for phenotypic, histochemical, and biochemical analyses.

# 17. Phenotypic evaluation and histochemical staining of transgenic poplars

Wild-type and *StCesA1*-overexpressing poplar plants were grown in a greenhouse for three months under controlled conditions at 25°C with a 16 h/8 h light/dark photoperiod. Plant height and basal stem diameter were measured. The 10th internodes were collected and fixed in formalin-acetic acid-alcohol (FAA) for more than 24 h. Fixed stem samples were sectioned using a vibratome (VT1000S; Leica, Wetzlar, Germany) at a thickness of 30 μm.

Stem cross-sections were stained with 2% phloroglucinol-HCl for lignin visualization and 0.01% Calcofluor White (18909; Sigma) for cellulose staining. Stained sections were observed under an Axio Imager A1 microscope. These histochemical analyses were used to compare lignin distribution, cellulose deposition, and xylem structure between wild-type and *StCesA1*-overexpressing plants.

# 18. Cellulose and lignin content determination

For biochemical quantification, stem segments from the 7th to 15th internodes were harvested, oven-dried at 65°C to constant weight, and ground into a homogeneous powder. Lignin content was measured using a lignin content kit (G0708W; Grace Biotechnology, Suzhou, China), based on acetylation of phenolic hydroxyl groups in lignin. Cellulose content was measured using a cellulose content kit (G0715W; Grace Biotechnology, Suzhou, China), based on hydrolysis and dehydration of cellulose into furfural compounds followed by colorimetric reaction with anthrone. All assays were performed according to the manufacturer’s instructions.

# 19. Biological replication and statistical analysis

All phenotypic, histochemical, and biochemical measurements of transgenic poplars included at least three independent biological replicates. Data are presented as mean +/- standard deviation (SD). For pairwise comparisons between a transgenic line and wild-type plants, statistical significance was evaluated using a two-tailed Student’s t-test. When multiple transgenic lines and wild-type plants were compared simultaneously, one-way analysis of variance (ANOVA) followed by Duncan’s multiple range test was used where appropriate, with *P* < 0.05 considered statistically significant. In figures, asterisks indicate significant differences, with **P* < 0.05 and ***P* < 0.01.
